# Clinical impact of endometrial cancer stratified by genetic mutational profiles, *POLE* mutation, and microsatellite instability

**DOI:** 10.1371/journal.pone.0195655

**Published:** 2018-04-16

**Authors:** Tomoko Haruma, Takeshi Nagasaka, Keiichiro Nakamura, Junko Haraga, Akihiro Nyuya, Takeshi Nishida, Ajay Goel, Hisashi Masuyama, Yuji Hiramatsu

**Affiliations:** 1 Department of Obstetrics and Gynecology, Okayama University Graduate School of Medicine, Dentistry and Pharmaceutical Sciences, Okayama, Japan; 2 Department of Clinical Oncology, Kawasaki Medical School, Kurashiki, Japan; 3 Center for Gastrointestinal Research, Center for Translational Genomics and Oncology, Baylor Scott & White Research Institute, Dallas, Texas, United States of America; 4 Charles A Sammons Cancer Center, Baylor University Medical Center, Dallas, Texas, United States of America; Ohio State University Wexner Medical Center, UNITED STATES

## Abstract

**Background:**

The molecular characterization of endometrial cancer (EC) can facilitate identification of various tumor subtypes. Although EC patients with *POLE* mutations reproducibly demonstrate better prognosis, the outcome of patients with microsatellite instability (MSI) remains controversial. This study attempted to interrogate whether genetic stratification of EC can identify distinct subsets with prognostic significance.

**Materials and methods:**

A cohort of 138 EC patients who underwent surgical resection with curative intent was enrolled. Sanger sequencing was used to evaluate mutations in the *POLE* and *KRAS* genes. MSI analysis was performed using four mononucleotide repeat markers and methylation status of the *MLH1* promoter was measured by a fluorescent bisulfite polymerase chain reaction (PCR). Protein expression for mismatch repair (MMR) proteins was evaluated by immunohistochemistry (IHC).

**Results:**

Extensive hypermethylation of the *MLH1* promoter was observed in 69.6% ECs with MLH1 deficiency and 3.5% with MMR proficiency, but in none of the ECs with loss of other MMR genes (*P* < .0001). MSI-positive and *POLE* mutations were found in 29.0% and 8.7% EC patients, respectively. Our MSI analysis showed a sensitivity of 92.7% for EC patients with MMR deficiency, and a specificity of 97.9% for EC patients with MMR proficiency. In univariate and multivariate analyses, *POLE* mutations and *MSI* status was significantly associated with progression-free survival (*P* = 0.0129 and 0.0064, respectively) but not with endometrial cancer-specific survival.

**Conclusions:**

This study provides significant evidence that analyses of proofreading *POLE* mutations and MSI status based on mononucleotide repeat markers are potentially useful biomarkers to identify EC patients with better prognosis.

## Introduction

Endometrial cancer (EC) is one of the most common gynecologic malignancy in the western world and Japan, and its prevalence has increased in recent years [1]. Lately, several important advances have been made in defining the molecular alterations that contribute towards endometrial tumorigenesis [2–10]. The Cancer Genome Atlas Research Network (TCGA) has provided new insights that ECs can be divided into four categories according to various genetic and epigenetic features: an ultramutated phenotype caused by *POLE* mutations, a hypermutator phenotype caused by the DNA mismatch repair deficiency (dMMR) leading to microsatellite instability (MSI), a copy number low phenotype, and a copy number high phenotype [2].

Among these alterations, the *POLE* gene is a catalytic subunit of DNA polymerase epsilon that is involved in nuclear DNA replication and repair. Hotspot mutations are located in the exonuclease domain of *POLE* (exons 9–14) which cause an ultramutated phenotype in colorectal and endometrioid tumors. Especially of EC cases, hotspot mutations in exon 9 (P286R and S297F) and exon 13 (V411L, L424V and L424I) were reported and EC patients with such *POLE* mutations demonstrate a better progression-free survival [2, 3, 10, 11].

MSI is caused by dMMR, which results in greatly increased rates of strand-slippage mutations, the so-called hypermutator phenotype compared with ECs harboring *POLE* mutations. Although the majority of ECs with dMMR are sporadic, 3% to 5% of cases develop disease because of inherited mutations in MMR genes (Lynch syndrome) [4]. Universal screening by evaluating tumor MSI status and MMR immunohistochemistry (IHC) has been widely adapted to screen Lynch syndrome, especially in patients with colorectal cancer [12, 13]. In addition to identifying potential germline mutation carriers, MMR analysis of colorectal and non-colorectal tumors is used as both a prognostic and a predictive approach for PD-1 targeted therapies [14, 15]. Although ECs with ultramutator phenotype consistently demonstrate better outcomes, patent survival in EC patients with hypermutated dMMR/MSI remain controversial [2, 3, 5, 9, 16–23].

In this retrospective study, we initially analyzed genetic mutations in the *POLE* gene, evaluated tumor MSI status, *MLH1* promoter methylation profile, and MMR expression status in all 138 ECs. Finally, we classified ECs according to the genetic profiles based on *POLE* mutations and MSI status to determine their precise relationship with various clinic-pathological features.

## Materials and methods

### Study participants

A cohort of 138 patients with EC resected at Okayama University Hospital (Okayama, Japan) from 2006 to 2009 was enrolled in this study. All patients underwent surgery followed by adjuvant chemotherapy and/or radiation if indicated. Institutional review board approval was granted by the ethics committee of the Okayama University, and written informed consent was obtained from all patients to use their tissues for research. The medical records of the patients were retrospectively explored and matched with clinical and pathological data. Standard post-treatment surveillance included serial physical examination with pap smears and computed tomography (CT) including positron emission tomography-computed tomography (PET-CT) every 3 to 6 months. Clinical data was abstracted from hospital records and included age at diagnosis, surgical International Federation of Gynecology and Obstetrics (FIGO) stage, adjuvant treatment and outcomes. Experienced gynecologic pathologists evaluated all cases for pathological information such as tumor grade, histologic subtype, depth of myometrial invasion, cervical stromal invasion, and lymphovascular space invasion (LVSI) and confirmed diagnoses.

### DNA extraction and bisulfite modification

We collected formalin-fixed, paraffin-embedded (FFPE) tissue specimens of primary EC from the cohort of 138 patients who had undergone surgery. DNA was extracted by the TaKaRa DEXPAT kit (Takara Bio Inc., Otsu, Japan) from EC tissue macro-dissected manually from FFPE tissue sections. Prior to sodium bisulfite modification, all genomic DNA was purified and concentrated by ethanol precipitation. Thereafter, genomic DNA was subjected to sodium bisulfite modification using the EZ DNA Methylation Kit (ZYMO Research, Irvine, CA).

### *POLE*, *KRAS*, and *BRAF* mutation analysis

Exon 9 and 13 in the *POLE* gene, *KRAS* exon 2 and *BRAF* exon 15 mutation status were analyzed in all 138 EC patients. Primer sequences for the *POLE* mutation analyses are shown in S1 Table. *KRAS* exon 2 and *BRAF* exon 15 mutation status were analyzed by using primer sets described previously [24]. The amplified PCR products were electrophoresed on an ABI 3100 Genetic Analyzer (Applied Biosystems, Foster City, CA, USA).

### MSI analysis

The MSI status was analyzed in all 138 ECs by using four mononucleotide repeat markers (BAT26, NR21, NR27, and CAT25), as described previously [25, 26]. When at least one or more mononucleotide repeat markers displayed MSI, tumors were defined to have an MSI phenotype and the tumors without MSI in the four mononucleotide repeat markers were defined to have a non-MSI phenotype according to our previous studies.

### Methylation analysis for the *MLH1* promoter

The *MLH1* gene promoter was divided into two regions (5’-region and 3’-region) as described previously [24, 27, 28]. The combined bisulfite restriction analysis was modified to measure methylation density quantitatively by a capillary sequencer. PCR products digested with *HhaI* or *RsaI* (New England BioLabs, Ipswitch, MA, USA) were loaded simultaneously onto an ABI 310R or 31000 Genetic Analyzer (Applied Biosystems, California, USA). Signals from individual PCR products were distinguished by the unique fluorescent PCR signal from each target and their fragment length, and the data were analyzed using GeneMapper software version 4.0 (Applied Biosystems, Foster City, CA, USA). In this study, the percentages of methylated *HhaI* or *RsaI* sites were calculated by determining the ratios between the *HhaI/RsaI*-cleaved PCR products and the total amount of PCR product in each locus and methylation positive was defined when the percentages of methylated *HhaI* or *RsaI* sites over 5.0%.

### MMR immunohistochemistry

We examined protein expression for MLH1, MSH2, PMS2, and MSH6 in 138 tumor tissues by immunohistochemical (IHC) staining using DAKO EnVision System-HRP polymer system kit (DakoCytomation California, Inc., Carpinteria, CA, USA). Staining was performed manually with FFPE specimens. Thin (5 μm) sections of representative blocks were deparaffinized and dehydrated using gradient solvents. Following antigen retrieval in the citrate buffer (pH 6.0), endogenous peroxidase was blocked with 3% H_2_O_2_. Thereafter, slides were incubated overnight in the presence of purified mouse monoclonal antibodies against MLH1 (clone G168-15, BD Pharmingen, San Diego, CA, USA; dilution 1:50), MSH2 (clone G219-1129, BD Pharmingen; dilution 1:200), PMS2 (clone A16-4, BD Pharmingen; dilution 1:200), and MSH6 protein (clone 44/MSH6, BD Pharmingen; dilution 1:100), respectively. A further incubation was performed with a secondary antibody and the avidin–biotin–peroxidase complex (Vector Laboratories, Burlingame, CA, USA) and then incubated with biotinyltyramide, followed by streptavidin–peroxidase. Diaminobenzidine was used as a chromogen and hematoxylin as a nuclear counterstain. Tumor cells were scored negative for MMR protein expression only if the epithelial cells within the tumor tissue lacked nuclear staining, while the surrounding stromal cells still showed positive staining. Samples showing proficiency in expression of all MMR proteins were defined as pMMR, and samples showing deficiency in at least one of the four MMR proteins were defined as dMMR.

### Statistical analyses

Statistical analyses were performed using JMP software (version 10.0; SAS Institute, Inc., Cary, NC, USA). First, methylation levels were analyzed as continuous variables. Next, the methylation status was analyzed as a categorical variable (positive, methylation level ≥ 5.0%; negative, methylation level < 5.0%). Categorical variables were compared by Fisher’s exact test. Endometrial cancer-specific survival (ECS) was calculated from the length of time from treatments including neo-adjuvant therapies or surgical resection to the date of death due to EC or last follow-up for censored patients. Progression-free survival (PFS) was defied as the time from surgical resection to recurrence or progression by CT and/or PET-CT routinely performed every 3 to 6 months. ECS and PFS were univariately estimated with the Kaplan–Meier method. Univariate and multivariate analyses for ECS and PFS were performed by Cox’s proportional hazard regression. Clinically accepted prognostic factors significant on univariate analysis were included in the model, including age, stage, tumor grade, histology, depth of invasion, LVSI, cervical stromal invasion, adjuvant treatment, *KRAS* status, and *POLE* mutation/MSI status. All reported *P* values were two-sided and a *P* value of less than 0.05 was considered statistically significant.

## Results

### Expression status of mismatch repair proteins

**Fig 1:**
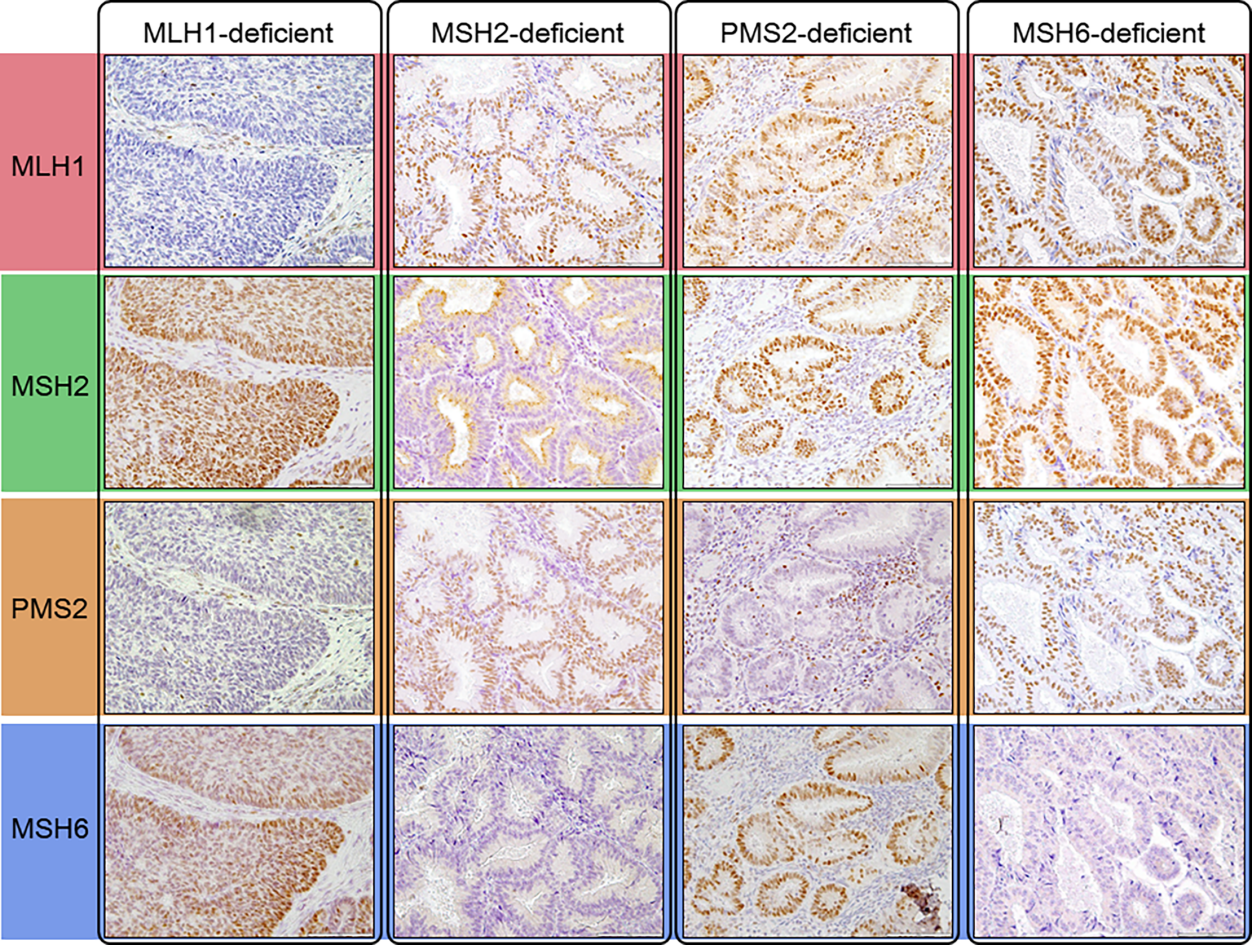
Representative examples of immunohistochemistry staining for the four MMR proteins. Tumors with MLH1-deficiency (dMLH1) show negative expression in both MLH1 and PMS2 IHC, those with MSH2-deficiency (dMSH2) show negative expression in both MSH2 and MSH6 IHC, with PMS2-deficiency (dPMS2) they show negative expression only in PMS2, and tumors with MSH6-deficiency show negative expression only in MSH6.

Clinicopathological findings and outcomes of 138 EC patients enrolled in this study are summarized in Table 1 and S1 Fig. In total, 123 endometrioid tumors (89.1%) and 15 others including clear cell and serous (10.9%) endometrial cancers were included. By FIGO staging criteria, stage I, II, III, and IV were 93 (67.4%), 11 (7.8%), 24 (17.4%), and 10 (7.2%), respectively. Expression status of the four MMR proteins (MLH1, MSH2, PMS2, and MSH6) was confirmed in all 138 EC tissues by IHC. Representative examples of IHC staining results are shown in Fig 1. By the IHC analysis, 97 tumors (70.3%) were classified as MMR-proficient (pMMR) and 41 (29.7%) as MMR-deficient (dMMR). Of 41 dMMR tumors, 23 (56.1% in dMMR) showed both MLH1- and PMS2-deficiency (dMLH1), 8 tumors (19.5%) both MSH2- and MSH6-deficiency (dMSH2), 8 tumors (19.5%) MSH6-deficiency alone (dMSH6), and 2 tumors (4.9%) PMS2-deficiency alone (dPMS2).

**Table 1:** Association between clinic-pathological features and EC patients stratified by genetic mutational profiles.

	All	*POLE-*mt	MSI	Non-MSI	P*
**Age**					0.3377
>60	60.1 (83)	75.0 (9)	65.0 (26)	55.8 (48)	
≤60	39.9 (55)	25.0 (3)	35.0 (14)	44.2 (38)	
**FIGO stage**					0.6536
I	67.4 (93)	83.3 (10)	65.0 (26)	66.3 (57)	
II	7.8 (11)	0 (0)	7.5 (3)	9.3 (8)	
III	17.4 (24)	16.7 (2)	22.5 (9)	15.1 (13)	
IV	7.2 (10)	0 (0)	5.0 (2)	9.3 (8)	
**Grade**					0.2204
G1	46.4 (64)	66.7 (8)	32.5 (13)	50.0 (43)	
G2	21.0 (29)	16.7 (2)	25.0 (10)	19.8 (17)	
G3	32.6 (45)	16.7 (2)	42.5 (17)	30.2 (26)	
**Histology**					0.2495
Endometrioid	89.1 (123)	100 (12)	92.5 (37)	86.1 (74)	
Others	10.9 (15)	0 (0)	7.5 (3)	13.9 (12)	
**Depth of myometrial invasion**					0.1825
Inner half	64.5 (89)	83.3 (10)	70.0 (28)	59.3 (51)	
Outer half or serosal	35.5 (49)	16.7 (2)	30.0 (12)	40.7 (35)	
**LVSI**					0.19
Negative	71.0 (98)	75.0 (9)	60.0 (24)	75.6 (65)	
Positive	29.0 (40)	25.0 (3)	40.0 (16)	24.4 (21)	
**Cervical stromal invasion**					0.3084
Negative	85.5 (118)	100 (12)	82.5 (33)	84.9 (73)	
Positive	14.5 (20)	0 (0)	35.0 (7)	15.1 (13)	
**BMI**					0.1437
<25	58.0 (80)	91.7 (11)	55.5 (22)	54.7 (47)	
25–30	21.7 (30)	0 (0)	30.0 (12)	20.9 (18)	
30–35	13.8 (19)	8.3 (1)	7.5 (3)	17.4 (15)	
>35	6.5 (9)	0 (0)	7.5 (3)	7.0 (6)	
**Any adjuvant treatment**					0.1909
No	46.4 (64)	66.7 (8)	37.5 (15)	47.7 (41)	
Yes	53.6 (74)	33.3 (4)	62.5 (25)	52.3 (45)	
***KRAS* status**					0.5335
Mutant	15.2 (21)	8.3 (1)	20.0 (8)	14.0 (12)	
Wild type	84.8 (117)	91.7 (11)	80.0 (32)	86.1 (74)	
***MLH1* 5'-region methylation**					< .0001
Methylated	30.4 (42)	16.7 (2)	65.0 (26)	16.3 (14)	
Unmethylated	69.6 (96)	83.3 (10)	35.0 (14)	83.7 (72)	
***MLH1* 3'-region methylation**					< .0001
Methylated	13.0 (18)	0 (0)	37.5 (15)	3.5 (3)	
Unmethylated	87.0 (120)	100 (12)	62.5 (25)	96.5 (83)	
***MLH1* methylation status**					< .0001
Extensively Methylated (both 5’- and 3’-region methylated)	13.0 (18)	0 (0)	37.5 (15)	3.5 (3)	
Partially Methylated (either 5’- or 3’-region methylated)	17.4 (24)	100 (2)	27.5 (11)	12.8 (11)	
Unmethylated	70.0 (96)	0 (10)	35.0 (14)	83.7 (72)	
**MMR expression status**					< .0001
MLH1-deficiency	16.7 (23)	0 (0)	55.0 (22)	1.2 (1)	
MSH2-deficiency	5.8 (8)	0 (0)	20.0 (8)	0 (0)	
PMS2-deficiency	1.4 (2)	0 (0)	5.0 (2)	0 (0)	
MSH6-deficiency	5.8 (8)	0 (0)	15.0 (6)	2.3 (2)	
MMR-proficiency	70.3 (97)	100 (12)	5.0 (2)	96.5 (83)	

LSVI and BMI denote lymphovascular space invasion and body mass index, respectively. MMR denotes mismatch repair.

*P values were calculated by chi-squire test.

### Association between methylation profiles of two discrete promoter regions in *MLH1* and *MLH1* protein expression

**Fig 2:**
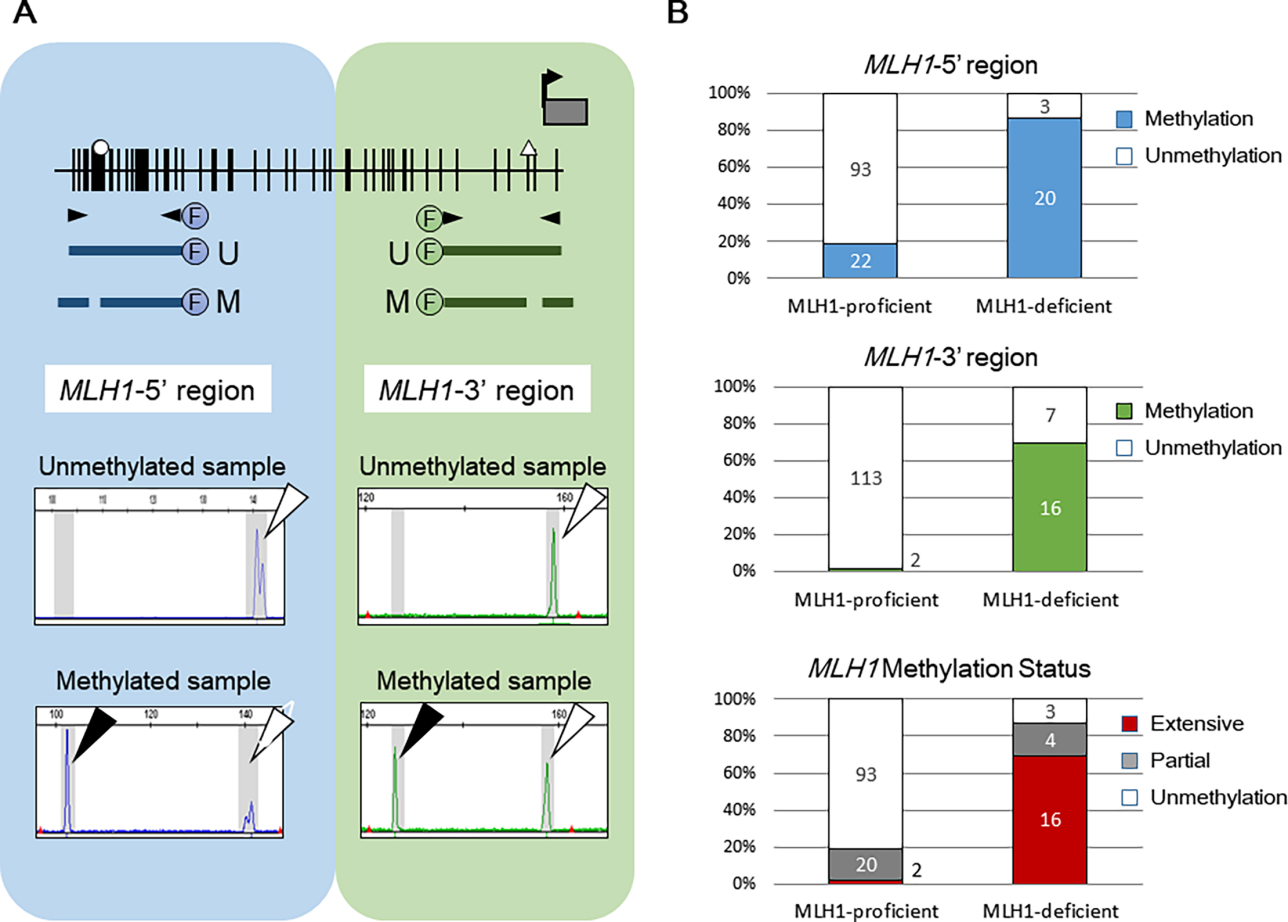
Methylation analysis of the promoter region in the *MLH1* gene. (A) Schematic depiction of two regions (5’-region and 3’-region) of the *MLH1* promoter for methylation and results of a panel of representative fluorescent bisulfite PCR following restriction enzyme analysis. Methylated samples had the new fragment cleaved by the restriction enzyme. (B) The frequencies of *MLH1* promoter methylation according to *MLH1* expression status. The top panel shows the results of the *MLH1*-5’ region, the middle panel shows the *MLH1*-3’ region and the bottom panel shows partial (i.e. only *MLH1*-5’ methylation) and extensive methylation (i.e. both *MLH1*-5’ and -3’ methylation).

In view of the published evidence that to inactivate MLH1 expression, extensive methylation towards whole promoter CpG region in the *MLH1* gene is required [24, 27, 29], we investigated the methylation status of both the 5’- and the 3’-regions of the *MLH1* promoter in a cohort of 138 ECs. Results of a panel of representative fluorescent bisulfite PCRs following restriction enzyme analysis are depicted in Panel A in Fig 2, and these results were analyzed with methylation as a continuous and a categorical variable. Partial methylation in *MLH1* (i.e. affecting the 5’-region only) was observed in 24 of 138 ECs (17.4%) and extensive methylation (i.e. affecting both the 5’-region and the 3’-region) was confirmed in 18 of 138 ECs (13.0%). Partial methylation in *MLH1* was observed in 13 of 97 (13.4%) pMMR-ECs, 4 of 23 (17.4%) ECs with dMLH1, 4 of 8 (50.0%) ECs with dMSH2, 2 of 8 (25.0%) ECs with dMSH6, and 1 of 2 (50.0%) ECs with dPMS2, whereas extensive methylation was detected in 2 of 97 (2.1%) ECs with pMMR, 16 of 23 (69.6%) ECs with dMLH1, none of the other dMMR (*P* < .0001, Panel B in Fig 2).

### *KRAS/BRAF* mutation status

Because sporadic MSI/dMMR phenotype in colorectal cancer is strongly associated with *BRAF* V600E mutation [24, 27]. We analyzed mutations in the *KRAS* and *BRAF* genes (Panel A in S2 Fig). In this cohort, no *BRAF* mutation were observed in exon 15 while *KRAS* mutations in exon 2 were present in 20 EC patients (14.5%), and the spectrum of relative frequency of individual mutations was 7 (35.0% in all *KRAS* mutant), 7 (35.0%), 2 (10.0%), 2 (10.0%), 1 (5.0%), and 1 (5.0%) for the G12D, G12V, G12A, G12C, G13D, and G13S mutations, respectively.

### *POLE* mutation status

As determination of ECs with *POLE* mutations was the first step in the stratification based upon their genomic features, we examined proofreading *POLE* mutations in exon 9 and 13 [2]. By conventional Sanger sequencing, a total of 12 ECs (8.7%) with *POLE* mutations was observed; the spectrum of relative frequencies of individual mutations was 7 (58.3% of *POLE* mutations) for P286R and 5 (41.7% of *POLE* mutations) for V411L, all of the *POLE* mutations we confirmed was exonuclease domain hotspot mutations (Panel B in S2 Fig).

### Association between MSI, *POLE* mutation, *MLH1* methylation, and MMR protein expression status

**Fig 3:**
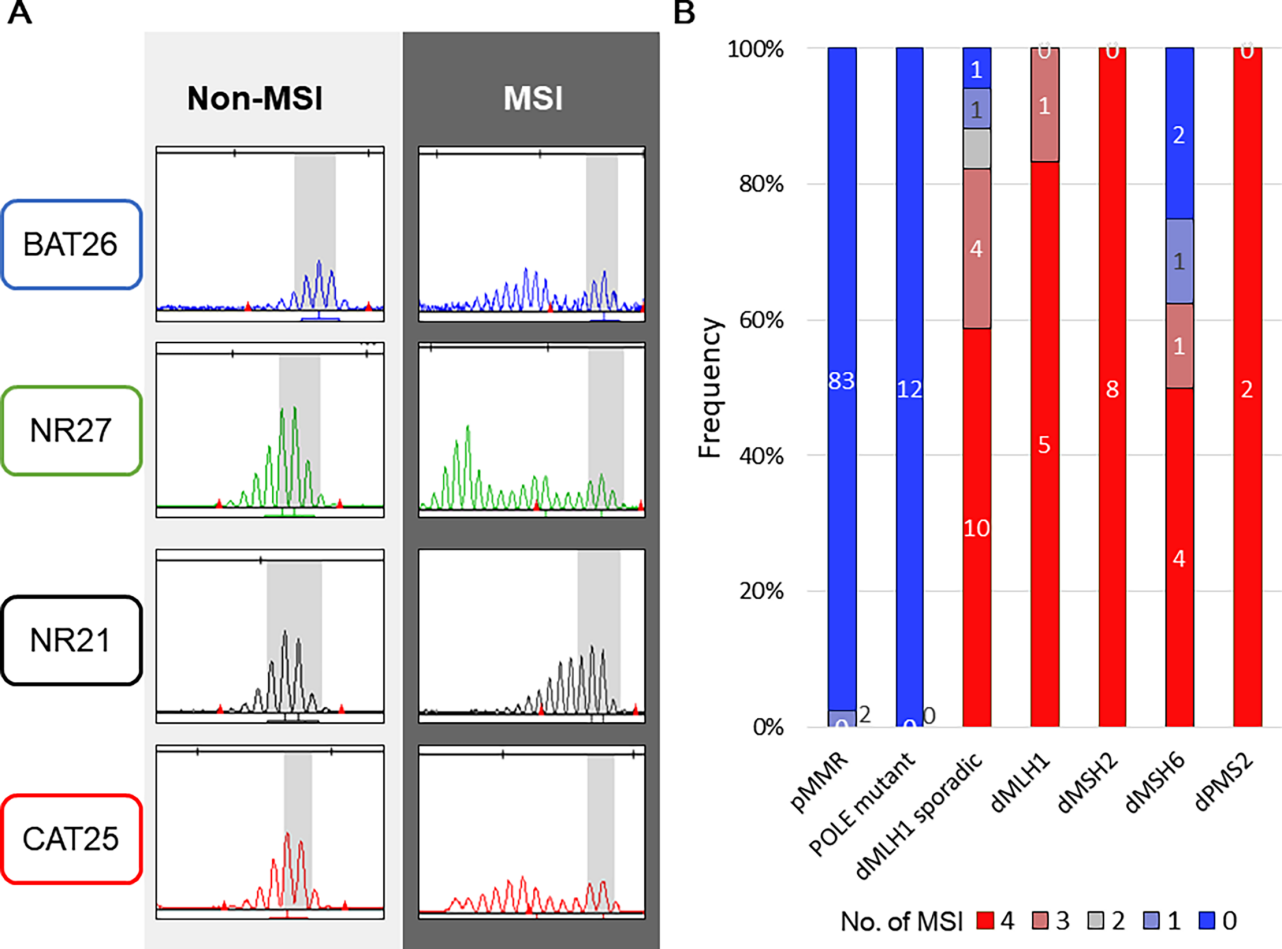
Detection of MSI and distribution of number of MSIs in 138 EC patients. (A) Example of MSI and non-MSI cases analyzed by four mononucleotide repeat markers (BAT26, NR21, NR27, and CAT25). (B) Association between MSI, *POLE* mutation, *MLH-1* promoter methylation and MMR protein expression. The number of mononucleotide repeat markers showing MSI are shown by color.

According to our previous studies[25, 26], tumors with MSI-positive (MSI) status were defined when at least one or more mononucleotide repeat markers displayed allelic variations and the tumors without MSI in the four mononucleotide repeat markers were defined as a non-MSI. By this criterion, we detected MSI tumors in 40 (29.0%) of the 138 EC patients. Panel A in Fig 3 shows representative examples of both pMMR and dMMR cases for each marker. By the four mononucleotide repeat markers, 92.7% (38 of 41) of MSI tumors showed dMMR, and 97.9% (95 of 97) of non-MSI tumors showed pMMR. Similar with previous studies, 12 ECs with *POLE* mutations defined as non-MSI by the four mononucleotide makers and positive staining in the four MMR proteins (Panel B in Fig 3). Regarding dMMR, only three cases did not show MSI signatures; one case was dMLH1 epigenetically silenced by *MLH1* promoter methylation and the other two cases were dMSH6.

### Clinical outcomes of EC patients with respect to stratification by mutational profiling

**Fig 4:**
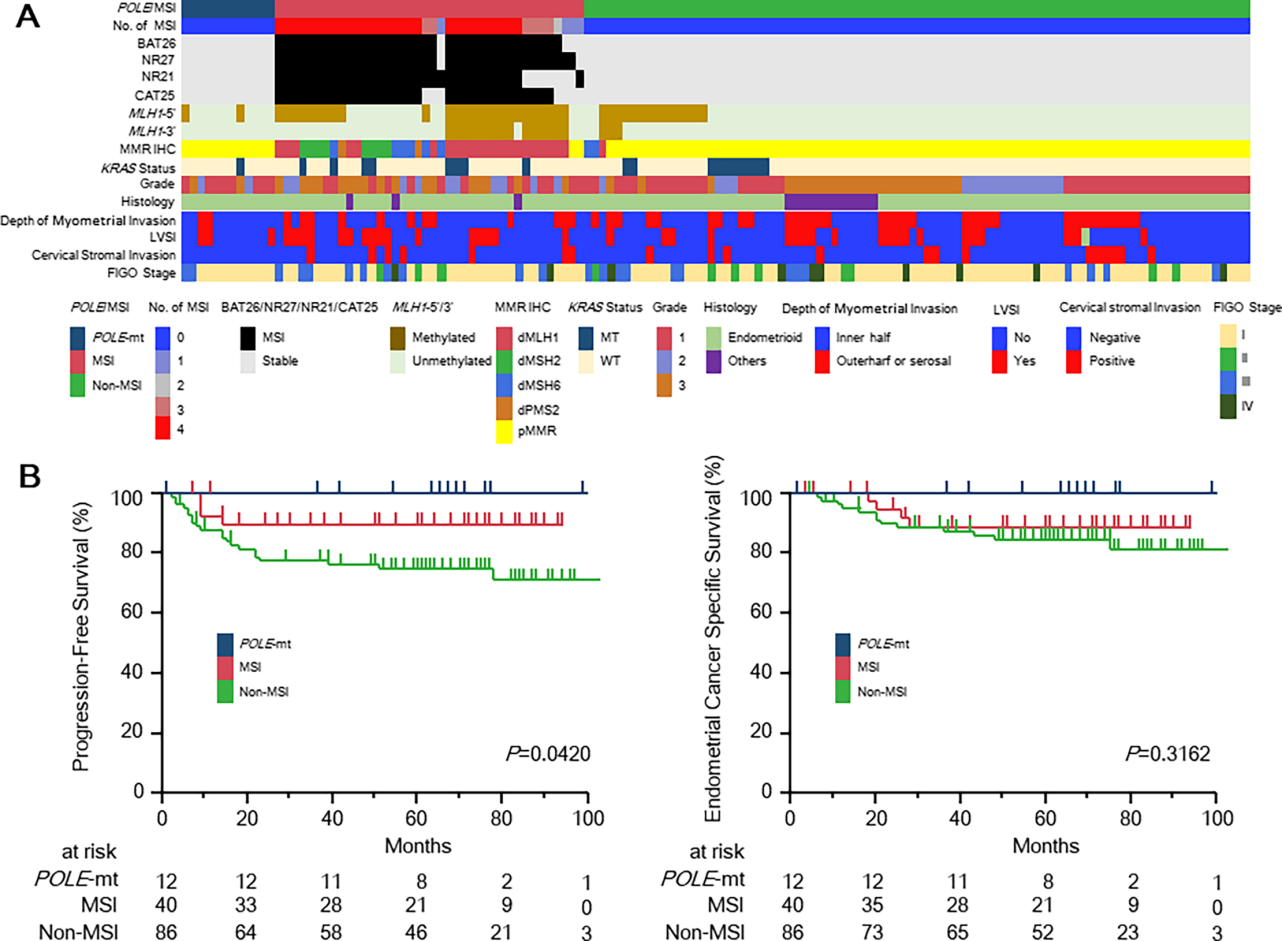
Molecular and clinic-pathological features of 138 ECs. (A) Molecular and clinic–pathological landscape of 138 ECs. Genetic analysis, focusing on frequent hotspot mutations in the POLE gene, and MSI status result in the identification of three molecular subgroups: (1) *POLE*-mutant, (2) MSI and (3) non-MSI. (B) Progression-free survival and endometrial cancer-specific survival of 138 EC patients stratified by genetic profiles. *P* values were calculated by the log-rank test.

Since *POLE* mutations and dMMR were mutually exclusive in our cohort, ECs were classified into the following three subsets; ECs with *POLE* mutations (*POLE*-mt), MSI, or non-MSI (Panel A in Fig 4). Table 1 shows the associations of clinicopathological features among the three subsets.

The median follow-up for PFS and ECS were 62 and 64 months, respectively (follow-up periods for both PFS and ECS: 1–105 months). For ECs with *POLE*-mutations, MSI and non-MSI, five-year PFSs were 100%, 89.5%, and 74.5% (*P* = 0.0420), five-year ECSs were 100%, 88.7%, and 84.5% (*P* = 0.3162), respectively (Panel B in Fig 4). In our cohort, there was no association between clinicopathologiclal findings and *POLE*-mutant/MSI status. In contrast, *MLH1* methylation status and MMR expression status were obviously associated with tumors with MSI. Finally, univariate and multivariate analysis are shown in Table 2. In the univariate analyses, FIGO stage, grade, histology, depth of myometrial invasion, LVSI, and *POLE/MSI* status were significantly associated with PFS. On the other hand, among those valuables, *KRAS* mutation status and *POLE/MSI* status was not associated with ECS. We next considered all variables to construct a multivariate model. The multivariate analysis for PFS demonstrated that *POLE/MSI* status, histology and adjuvant therapy are significantly associated with PFS, so these are considered as the prognostic factors. Again, the multivariate analysis for ECS demonstrated that only histology is associated with ECS.

**Table 2:** Univariate and multivariate outcome analyses of 138 EC patients.

Factor	PFS				OS			
	Univariate		Multivariate		Univariate		Multivariate	
	Risk Ratio (95%CI)	*P*	Risk Ratio (95%CI)	*P*	Risk Ratio (95%CI)	*P*	Risk Ratio (95%CI)	*P*
**Age**		0.0781		0.5093		0.5223		0.9695
>60 (vs <60)	2.05 (0.92–4.60)			1.47 (0.46–4.53)		1.38 (0.50–3.63)			1.03 (0.23–4.42)	
**FIGO stage**		**< .0001**		0.053		**< .0001**		0.0522
II (vs I)	1.31e-8 (0–5.21)	0.1795		7.4e-10 (0–0.94)	0.0444	8.14e-9 (0–5.34)	0.3494		1.51e-9 (0–2.90)	0.1102
III (vs I)	6.78 (2.05–25.9)	0.0002		2.16 (0.61–7.76)	0.2267	8.21 (2.46–31.5)	0.0007		5.04 (1.16–24.0)	0.1764
IV (vs I)	13.95 (3.99–54.6)	< .0001		1.43 (0.31–6.23)	0.6401	20.7 (5.89–81.4)	< .0001		5.39 (1.18–27.6)	0.1524
III (vs II)	9.89e+8 (2.73-∞)	0.0026		2.92e+9 (2.41-∞)	0.0065	1.01e+9 (1.71-∞)	0.0014		4.44e+9 (2.03-∞)	0.0124
IV (vs II)	2.27e+9 (5.93-∞)	0.0001		1.93e+9 (1.40-∞)	0.0274	2.55e+9 (4.21-∞)	0.0008		4.57 (1.98-∞)	0.014
IV (vs III)	2.30 (0.83–6.01)	0.1048		0.66 (0.17–2.30)	0.5211	2.52 (0.81–7.65)	0.1076		1.03 (0.23–4.47)	0.9689
**Grade**		**< .0001**		0.2022		**0.0009**		0.678
2 (vs 1)	2.17 (0.40–11.7)	0.3482		2.52 (0.43–14.7)	0.2863	3.23 (0.54–24.6)	0.194		2.32 (0.35–19.2)	0.3784
3 (vs 1)	10.9 (3.71–46.5)	< .0001		3.25 (0.88–15.6)	0.0772	9.87 (2.69–63.4)	0.0002		1.61 (0.30–12.1)	0.5882
3 (vs 2)	5.03 (1.71–21.4)	0.002		1.29 (0.35–6.25)	0.7165	3.05 (0.97–13.4)	0.0572		0.69 (0.15–3.58)	0.6365
**Histology**		**0.0002**		**0.0365**		**0.0005**		**0.0176**
Others (vs Endometrioid)	6.44 (2.60–14.7)			3.78 (1.09–13.0)		8.00 (2.73–21.3)			7.11 (1.42–38.1)	
**Depth of myometrial invasion**		**0.0029**		0.7137		**0.0275**		0.7224
Outer half or serosal (vs Inner half)	3.34 (1.51–7.70)			1.23 (0.41–3.75)		2.94 (1.13–8.12)			1.29 (0.30–5.50)	
**LVSI**		**< .0001**		0.0997		**0.0013**		0.6327
Yes (vs no)	6.39 (2.84–15.7)		2.93 (0.82–11.2)		4.93 (1.87–14.3)		1.44 (0.34–6.37)	
**Cervical stromal invasion**		0.174		0.2939		0.0908		0.2288
Yes (vs no)	1.97 (0.72–4.68)		2.16 (0.51–9.41)		2.66 (0.84–7.21)		2.51 (0.55–12.0)	
**Any adjuvant therapy**		**< .0001**		**0.0466**		**0.0002**		0.1245
Yes (vs no)	11.0 (3.26–68.8)		4.59 (1.02–32.6)		14.3 (2.92–258)		4.74 (0.68–95.9)	
***KRAS* status**		0.5299		0.9557		0.5792		0.7977
Mutant (vs wild-type)	0.69 (0.16–2.00)		1.05 (0.20–7.89)		0.67 (0.11–2.39)		1.34 (0.12–10.1)	
***POLE*/MSI status**		**0.0129**		**0.0064**		0.1477		0.1412
MSI (vs *POLE*-mt)	2.92e+8 (0.496-∞)	0.1438	1.44e+8 (0.12-∞)	0.405	4.27e+8 (0.52-∞)	0.4558	1.72e+8 (0.12-∞)	0.4147
Non-MSI (vs *POLE*-mt)	7.68e+8 (1.599-∞)	0.0144	7.33e+9 (0.79-∞)	0.0762	6.45e+8 (0.93-∞)	0.0583	4.81e+8 (0.39∞)	0.1843
Non-MSI (vs MSI)	2.63 (1.00–9.01)	0.05	5.10 (1.63–19.7)	0.0042	1.51 (0.53–5.36)	0.4558	2.80 (0.81–11.7)	0.1048

LSVI and MSI denote lymphovascular space invasion and microsatellite instability, respectively.

## Discussion

EC had historically been categorized into two pathogenic subtypes; type I and type II [30]. This classification lacks sufficient discriminative ability to categorize tumors or to guide the treatment decision of EC patents [31, 32]. Currently, all major risk-stratification systems for EC patients, the treatment recommendations are based on a combination of histological type, stage, and grade [33–35]. However, it has been demonstrated that these systems lack the discriminative ability to determine outcomes [36].

Several studies have performed molecular characterization of ECs and have identified mutational profiles to help distinguish EC subtypes [2–8]. The most comprehensive molecular study of EC to date has been from the TCGA, which included a combination of whole-genome sequencing, exome sequencing, MSI analyses, copy number analyses, and proteomics [2]. Molecular information was used to classify 232 patients with EC into four groups—*POLE*-mt, MSI, copy number low, and copy number high—which were correlated with PFS [2]. However, it would be cost-prohibitive and impractical to apply the range and extent of genomic and molecular tests used in the TCGA study to patients in a clinical setting.

ECs with proofreading *POLE* mutations showed better prognosis [2, 3, 10, 11]. We examined *POLE* mutations in exons 9 and 13. By the cBioPortal FOR CANCER GENOMICS (http://www.cbioportal.org/index.do) website, a total of 92 *POLE* mutations including 46 duplicate mutations in patients with multiple samples were found in EC (TCGA, Nature 2013 and Provisional cohorts). Among them, 32 mutations were considered to be pathogenic mutations; P286R and S297F located in exon 9 were found in 16 and 2 ECs, respectively; V411L, L424V and L424I located in exon 13 were found in 10, 2 and 2 ECs respectively (Panel C in S2 Fig). Of our 138 ECs, only P286R and V411L were detected in 7 and 5 ECs.

Our study attempted to determine whether genetic stratification of ECs can effectively identify distinct subsets with prognostic significance. Interestingly, we found highly significant and clinically relevant differences in relapse and survival rates between genetically stratified subgroups. Thus, our classification by the set of two mutational profiles, *POLE* mutation and MSI status, is rather easy to practical for routine clinical practice. As ECs consist of a heterogeneous group of tumors with diverse molecular alterations, our analyses further support previous studies that showed an association of *POLE* proofreading mutations with favorable prognosis [2, 3, 5, 10, 37, 38].

Defective DNA mismatch repair represents one of the most frequent molecular defects in EC, and tumors with such defects are readily identifiable through MSI analysis [39]. In this retrospective cohort, the population with MSI tumors was 29.0%, consistent with other studies [2, 3, 5, 16, 17, 39–41]. In this study we used the four mononucleotide repeat markers for the detection of MSI phenotype. By this MSI assay, tumors could be divided into the two phenotype; MSI and non-MSI, as we reported previously [25, 26]. Historically, MSI status was evaluated by the conventional MSI assay recommended by National Cancer Institute (NCI) workshop by the use of a reference panel of five markers: two mononucleotide-repeat markers (BAT26 and BAT25) and three dinucleotide repeat markers (D2S123, D5S346, and D17S250) [42]. By using this NCI recommended maker panel, tumors were divide into the three subtype; MSI-high, MSI-low and microsatellite stable (MSS). MSI-high tumors were always demonstrated dMMR and displayed MSI in almost of the five repeat makers irrespective of type of microsattelite markers, such as mononucleotide or dinucleotide repeat markers [26, 43, 44]. In contrast, MSI-low tumors showed pMMR and one or two sifted microsartellite markers mainly in di-nucleotide markers, not common in mononucleotide markers [43]. Based on those background, a pentaplex PCR system, as well as our MSI assay based on mononucleotide markers was developed to detect MMR deficient tumors [25, 26, 44].

The *MLH1* gene has a large CpG island within its promoter that clearly divides it into at least 2 discrete regions of methylation (Panel A in Fig 2). The methylation pattern is not homogeneous among various CpG sites within a CpG island. Deng et al. examined the methylation status of 3 regions (A, B and C) in the *MLH1* promoter, and compared the methylation status to the gene expression in 24 cell lines and concluded that only the C region was associated with the loss of gene expression [45]. In particular, the methylation in regions A and B (the 5’-region in this study) occurs in normal mucosa, and may spread toward region C (the 3’-region in this study) during tumor progression in colorectal cancer [24, 27, 46]. In ECs, extensive methylation (i.e. affecting both the 5’-region and the 3’-region) in the *MLH1* promoter region was detected in 69.6% of ECs with dMLH1 (dMMR by epigenetic alteration) and in none of ECs with other dMMR (dMMR probably by MMR mutations).

Recently, McMeekin et al. demonstrated dMMR caused by an epigenetic alteration (*MLH1* methylation) showed relatively worse prognosis compared with dMMR caused by probable MMR mutations or pMMR in large clinical cohorts [4]. In contrast, in line with few previous studies [2, 3, 5, 16, 23], our cohort demonstrated that ECs with MSI features (implicating dMMR) were associated with a reduced risk of recurrence and distant metastases (Table 2 and Fig 4). When we divided ECs with dMMR into two subclasses, dMMR by epigenetic alteration and probable MMR mutations, ECs with dMMR by epigenetic alteration showed relatively better outcome compared with ECs with dMMR by probable MMR mutations (data not shown).

Our cohort demonstrated that ECs with *POLE* mutations would have the better outcome among our three subsets. Indeed, the study by McMeekin et al lacks to stratify ECs with *POLE* mutations. Therefore, ECs with *POLE* mutations are lost in ECs with pMMR subclass, having a possibility to make clinical outcome of ECs with pMMR better.

Similar to our results, Stelloo et al demonstrated that ECs with *POLE* mutations and MSI showed better prognosis compared with other ECs by analyzing their large cohort obtained from three clinical trials (PORTEC-1, -2 and -3) and subclassifying EC patients with neither *POLE* mutation nor MSI into two subtypes according to *p53* mutational status; ECs with p53 mutations (p53-mutant) and no specific molecular profile (NSMP) [3, 5]. Interestingly, the cohort of earlier grades (86.8% was Grade 1/2; PORTEC-1 and -2) showed better prognosis in the NSMP group compared with p53-mutant, whereas in the cohort of advanced grades (84.5% was Grade 3; PORTEC-3), NSMP had a worse prognosis similar to p53-mutant [3, 5]. Thus, although the prognostic character of NSMP varied, ECs with *POLE* mutations and MSI constantly showed a better prognosis than the other subtypes.

This study provides robust genetic analyses that can easily be implemented in prospective studies and clinical practice. Especially, we adhere to stratifying only by reproducible genetic analyses that are easily accessible for daily clinical practice. MSI analyses have some limitations for detecting dMMR tumors. In our previous study, although our data suggested that a marker panel consisting of BAT26, NR21 and NR27 markers was more accurate in detecting CRCs with dMMR, we showed that the use of mono-markers missed identifying 3 of 8 (47%) CRCs with dMSH6 [25]. Thus, we added a mononucleotide repeat marker, CAT25, to the three mononucleotide marker panel (BAT26, NR21 and NR27) to try to increase the sensitivity for detecting tumors with dMMR [26].

## Conclusions

In conclusion, we acknowledge that this study has some limitations. For instance, the number of analyzed samples was relatively small and from a retrospective cohort in a single hospital. However, this study provides robust genetic analyses that can easily be implemented in prospective studies and clinical practice. Although intratumor heterogeneity may interfere with prediction of the patient's tumor genetic profile, our data suggest that analyses of proof reading *POLE* mutations and MSI by mononucleotide markers will be useful as biomarkers for identifying patients who have a good prognosis and may not require intensive postoperative radiotherapy or even chemotherapy.

## Supporting information

S1 Table Primer sequence for POLE mutations. (XLSX)

S1 Fig Progression free survival and endometrial cancer specific survival of 138 EC patients. Progression free survival and endometrial cancer specific survival of 138 EC patients stratified by tumor grade (**A**), FIGO stage (**B**) tumor type (**C**). and *P* values were calculated by log-rank test.
(TIF)

S2 Fig *KRAS* and *POLE* mutation analyses. (A) Examples of *KRAS* mutations in EC specimens. (B) Examples of *POLE* mutations in EC specimens.
(TIF)
