# Supplementary figures and images for: Clinical impact of endometrial cancer stratified by genetic mutational profiles, POLE mutation, and microsatellite instability
Source: PLoS One. 2018 Apr 16;13(4):e0195655. doi: 10.1371/journal.pone.0195655 (PMC5901772; doi:10.1371/journal.pone.0195655)

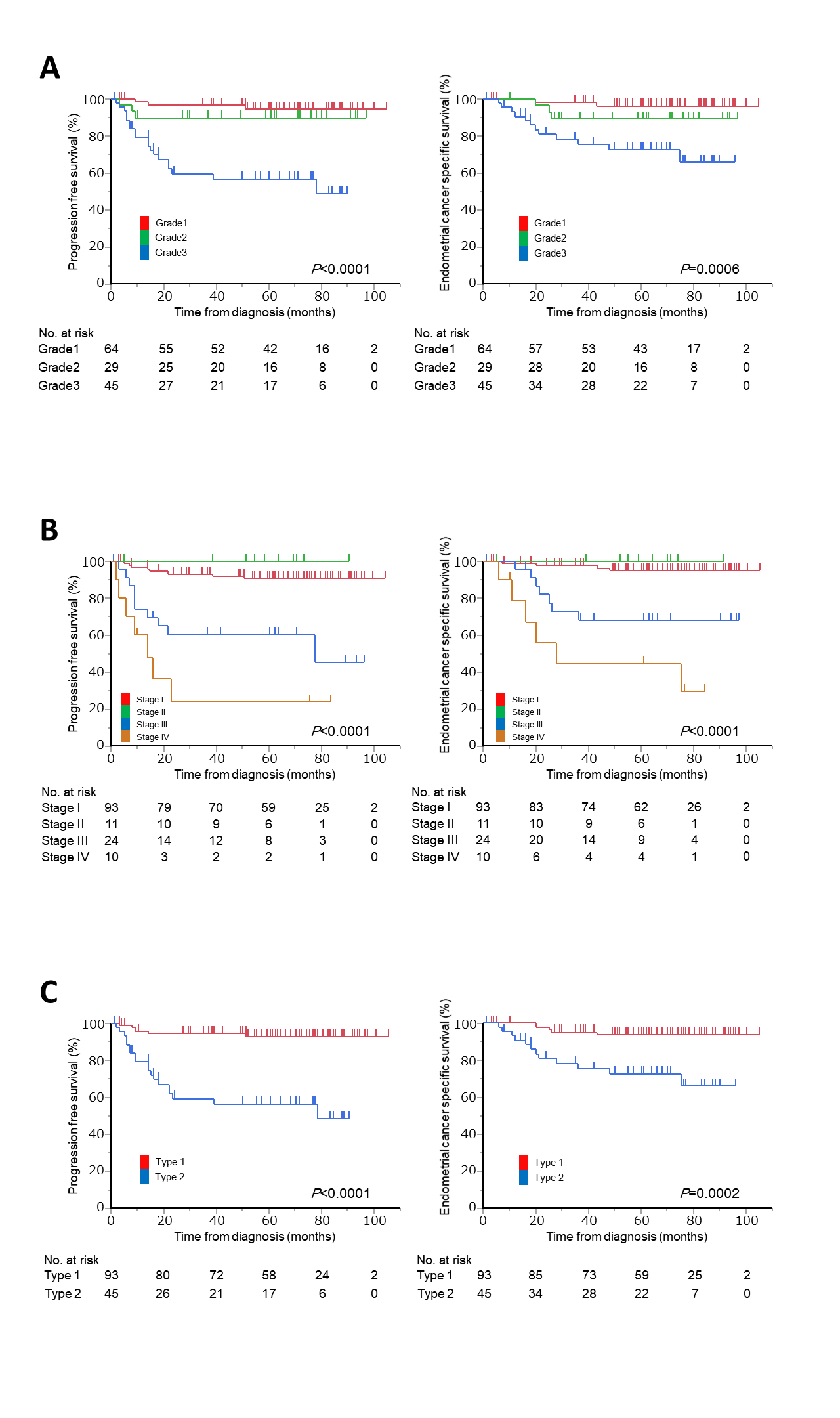

Supplement: S1 Fig — Progression free survival and endometrial cancer specific survival of 138 EC patients stratified by tumor grade (A), FIGO stage (B) tumor type (C). and P values were calculated by log-rank test. (TIF) [file pone.0195655.s002.tif]

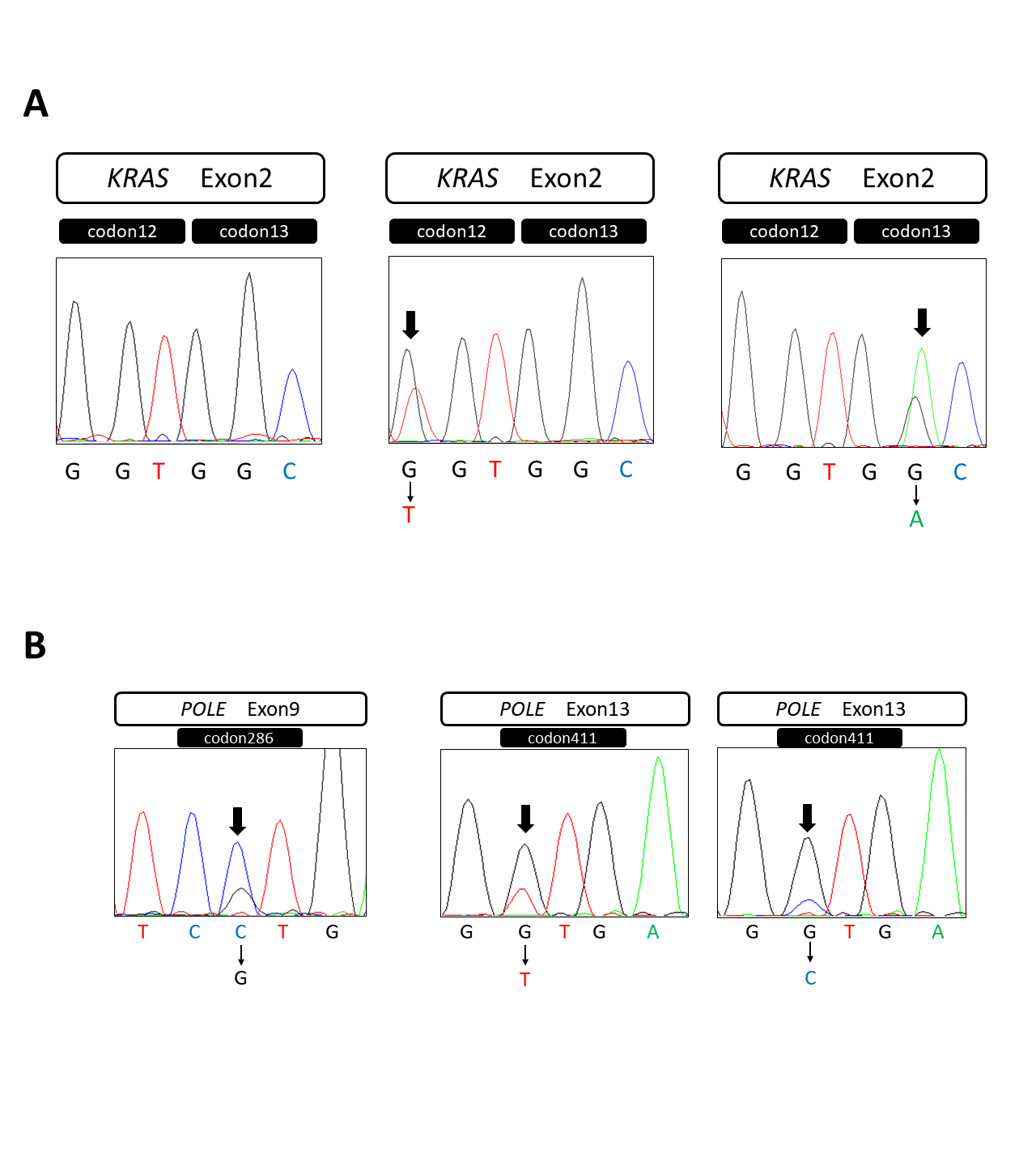

Supplement: S2 Fig — (A) Examples of KRAS mutations in EC specimens. (B) Examples of POLE mutations in EC specimens. (TIF) [file pone.0195655.s003.tif]
